# A guide to selecting high-performing antibodies for Serine/threonine-protein phosphatase 2A 56 kDa regulatory subunit delta isoform (PPP2R5D) for use in Western Blot, immunoprecipitation and immunofluorescence

**DOI:** 10.12688/f1000research.145146.1

**Published:** 2024-01-03

**Authors:** Riham Ayoubi, Maryam Fotouhi, Charles Alende, Kathleen Southern, Carl Laflamme, NeuroSGC/YCharOS/EDDU collaborative group, ABIF consortium

**Affiliations:** 1 Department of Neurology and Neurosurgery, Structural Genomics Consortium, The Montreal Neurological Institute, McGill University, Montreal, Québec, H3A 2B4, Canada

**Keywords:** Uniprot ID Q14738, PPP2R5D, Serine/threonine-protein phosphatase 2A 56 kDa regulatory subunit delta isoform, antibody characterization, antibody validation, Western Blot, immunoprecipitation, immunofluorescence

## Abstract

Protein phosphatase 2A is a serine/threonine phosphatase with activity dependent on an associated regulatory subunit, serine/threonine-protein phosphatase 2A 56 kDa regulatory subunit delta (δ) isoform (PPP2R5D). PPP2R5D is the δ isoform in the B56 family of regulatory subunits. Abundantly expressed in the brain and involved in a broad range of cellular processes, PPP2R5D plays an essential role in modulating key neuronal pathways and signalling. Pathogenic mutations in the *PPP2R5D* gene are linked to clinical symptoms characterized by neurodevelopmental delay, intellectual disability, and autism spectrum disorders. The etiology of these genetic disorders remains unknown, which can partly be due to the lack of independently characterized antibodies.

Here we have characterized six PPP2R5D commercial antibodies for Western Blot, immunoprecipitation, and immunofluorescence using a standardized experimental protocol based on comparing read-outs in knockout cell lines and isogenic parental controls. These studies are part of a larger, collaborative initiative seeking to address antibody reproducibility by characterizing commercially available antibodies for human proteins and publishing the results openly as a resource for the scientific community. While use of antibodies and protocols vary between laboratories, we encourage readers to use this report as a guide to select the most appropriate antibodies for their specific needs.

## Introduction

Protein phosphatase 2A (PP2A) is a ubiquitously expressed serine/threonine phosphatase that regulates various cellular functions including cell metabolism, cell cycle, DNA replication, transcription and translation, signal transduction, cell mobility and apoptosis. ^1^^–^^4^ PP2A has a heterotrimeric form consisting of 3 subunits: catalytic, scaffold and regulatory. ^5^ The specificity and activity of the PP2A enzyme is regulated by the associated regulatory subunit,which controls substrate selectivity and activity of PP2A. ^6^^–^^9^

The PP2A-B56 family of the regulatory subunit has 5 isoforms; α, β, γ, δ, and ε. The *PPP2R5D* gene encodes serine/threonine-protein phosphatase 2A 56 kDa regulatory subunit delta (δ) isoform (PPP2R5D). ^9^^,^^10^ This δ isoform is highly expressed in neuronal tissues and is crucial for cell cycle regulation. It controls the ability of PP2A to negatively regulate PI3K/AKT signalling pathways and modulates glycogen synthase kinase-3 β and cyclin-dependent kinase 5 activities by controlling tau phosphorylation. ^8^^,^^11^ Missense mutations in *PPP2R5D* have been linked to neurodevelopmental disorders such as neurodevelopmental delay, ^12^ intellectual disability ^13^^–^^15^ and autism spectrum disorder. ^16^^–^^20^ The majority of these mutations lead to Jordan’s syndrome, an autosomal dominant illness associated with intellectual disabilities and autism spectrum disorders. ^20^ Exploring the intricate network involving PPP2R5D as it impacts cell cycle regulation in neuronal processes could significantly enhance the potential for uncovering novel treatments for neurodevelopmental disorders. Further research is required and would be facilitated with the availability of high-quality antibodies.

This research is part of a broader collaborative initiative in which academics, funders and commercial antibody manufacturers are working together to address antibody reproducibility issues by characterizing commercial antibodies for human proteins using standardized protocols, and openly sharing the data. ^21^^–^^23^ Here, we evaluated the performance of six commercially-available antibodies for PPP2R5D in Western Blot, immunoprecipitation and immunofluorescence using a knockout based approach. This article serves as a valuable guide to help researchers select high-quality antibodies for their specific needs, facilitating the biochemical and cellular assessment of PPP2R5D properties and function.

## Results and discussion

Our standard protocol involves comparing readouts from wild-type (WT) and knockout (KO) cells. ^24^^,^^25^ The first step is to identify a cell line(s) that expresses sufficient endogenous levels of a given protein to generate a measurable signal. To this end, we examined the DepMap transcriptomics database to identify all cell lines that express the target at levels greater than 2.5 log _2_ (transcripts per million “TPM” + 1), which we have found to be a suitable cut-off (Cancer Dependency Map Portal, RRID:SCR_017655). Commercially available HAP1 cells expressed the PPP2R5D transcript at RNA levels above the average range of cancer cells analyzed. Parental and *PPP2R5D* KO HAP1 cells were obtained from Horizon Discovery ( Table 1).

**Table 1.** Summary of the cell lines used.

Institution	Catalog number	RRID (Cellosaurus)	Cell line	Genotype
Horizon Discovery	C631	CVCL_Y019	HAP1	WT
Horizon Discovery	HZGHC003155c003	CVCL_TG11	HAP1	*PPP2R5D* KO

**Figure 1.**
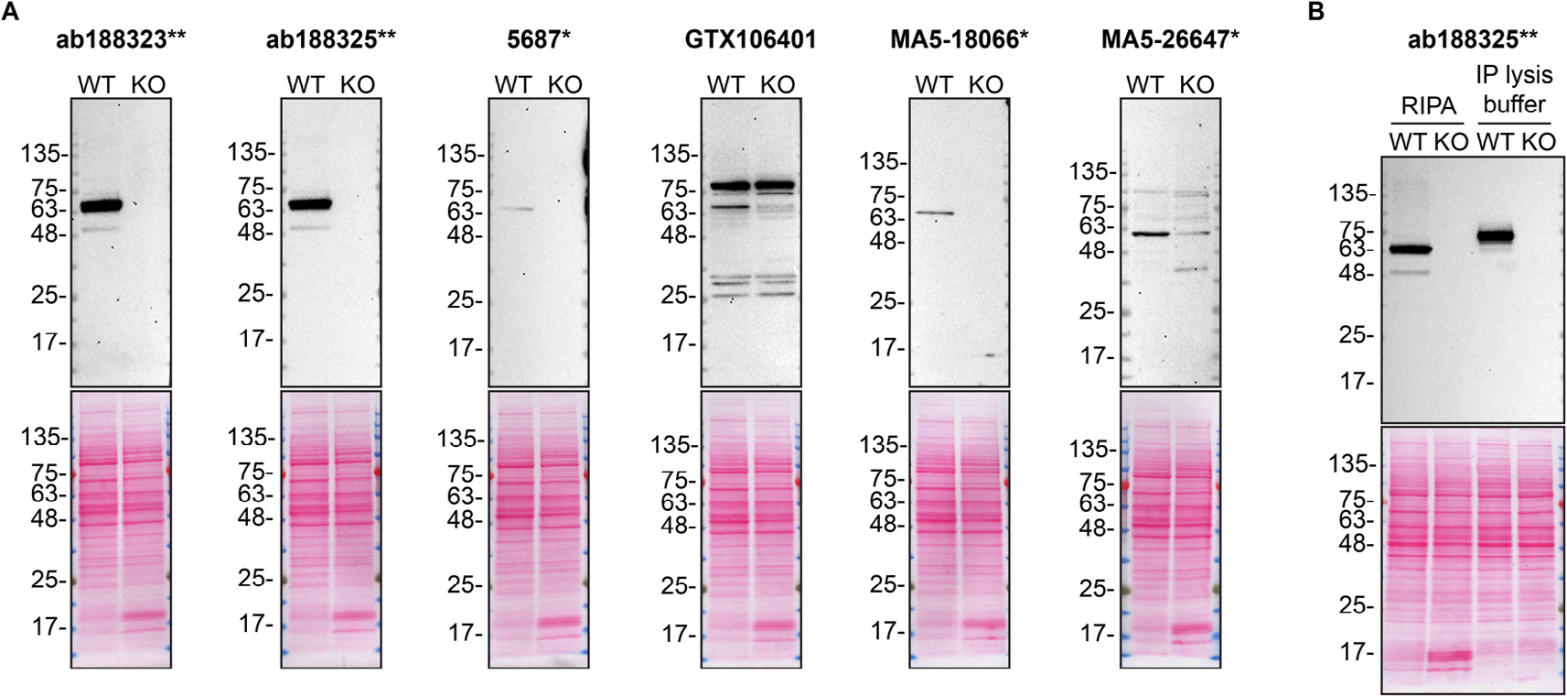
PPP2R5D antibody screening by Western Blot. A) Lysates of HAP1 WT and *PPP2R5D* KO were prepared in RIPA buffer, and 30 μg of protein were processed for Western Blot with the indicated PPP2R5D antibodies. The Ponceau stained transfers of each blot are shown. Antibody dilutions were chosen according to the recommendations of the antibody supplier. Exceptions were given for antibodies 5687*, MA5-18066* and MA5-26647*, which were titrated to the corresponding dilutions found below, as the signals were too weak when following the supplier’s recommendations. Antibody dilution used: ab188323** at 1/10 000, ab188325** at 1/10 000, 5687* at 1/200, GTX106401 at 1/500, MA5-18066* at 1/200, MA5-26647* at 1/200. B) The observed molecular weight of PPP2R5D varies according to the lysis buffer used. HAP1 WT and *PPP2R5D* KO lysates were prepared in RIPA or IP lysis buffer and the molecular weight of PPP2R5D was assessed by WB. The composition of lysis buffers is indicated in the Methods section. PPP2R5D ran primarily at ~65 kDa or ~75 kDa when lysates were prepared with RIPA or IP lysis buffer, respectively. ab188323** was used at 1/10 000 for Western Blot.

For Western Blot experiments, we resolved proteins from WT and *PPP2R5D* KO cell extracts and probed them side-by-side with all antibodies in parallel ( Figure 1).

**Figure 2.**
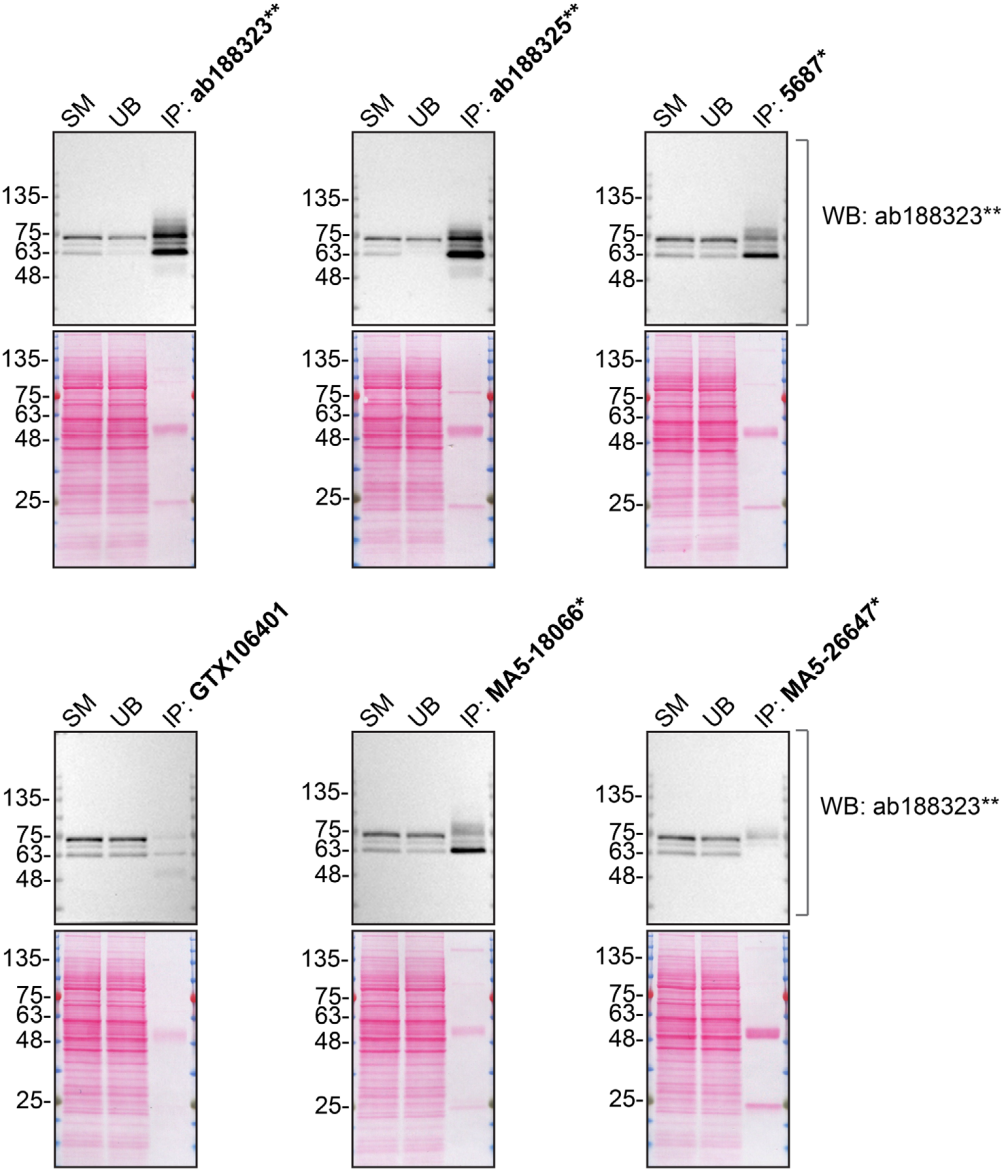
PPP2R5D antibody screening by immunoprecipitation. HAP1 lysates were prepared, and immunoprecipitation was performed using 2.0 μg of the indicated PPP2R5D antibodies pre-coupled to Dynabeads protein A or protein G. Samples were washed and processed for Western Blot with the indicated PPP2R5D antibody. For Western Blot, ab188323** was used at 1/5000. The Ponceau stained transfers of each blot are shown. SM=4% starting material; UB=4% unbound fraction; IP=immunoprecipitate. *Monoclonal antibody, **Recombinant antibody.

As per our standard procedure, we next used the antibodies to immunoprecipite PPP2R5D from HAP1 cell extracts. The performance of each antibody was evaluated by detecting the PPP2R5D protein in extracts, in the immunodepleted extracts and in the immunoprecipitates ( Figure 2).

**Figure 3.**
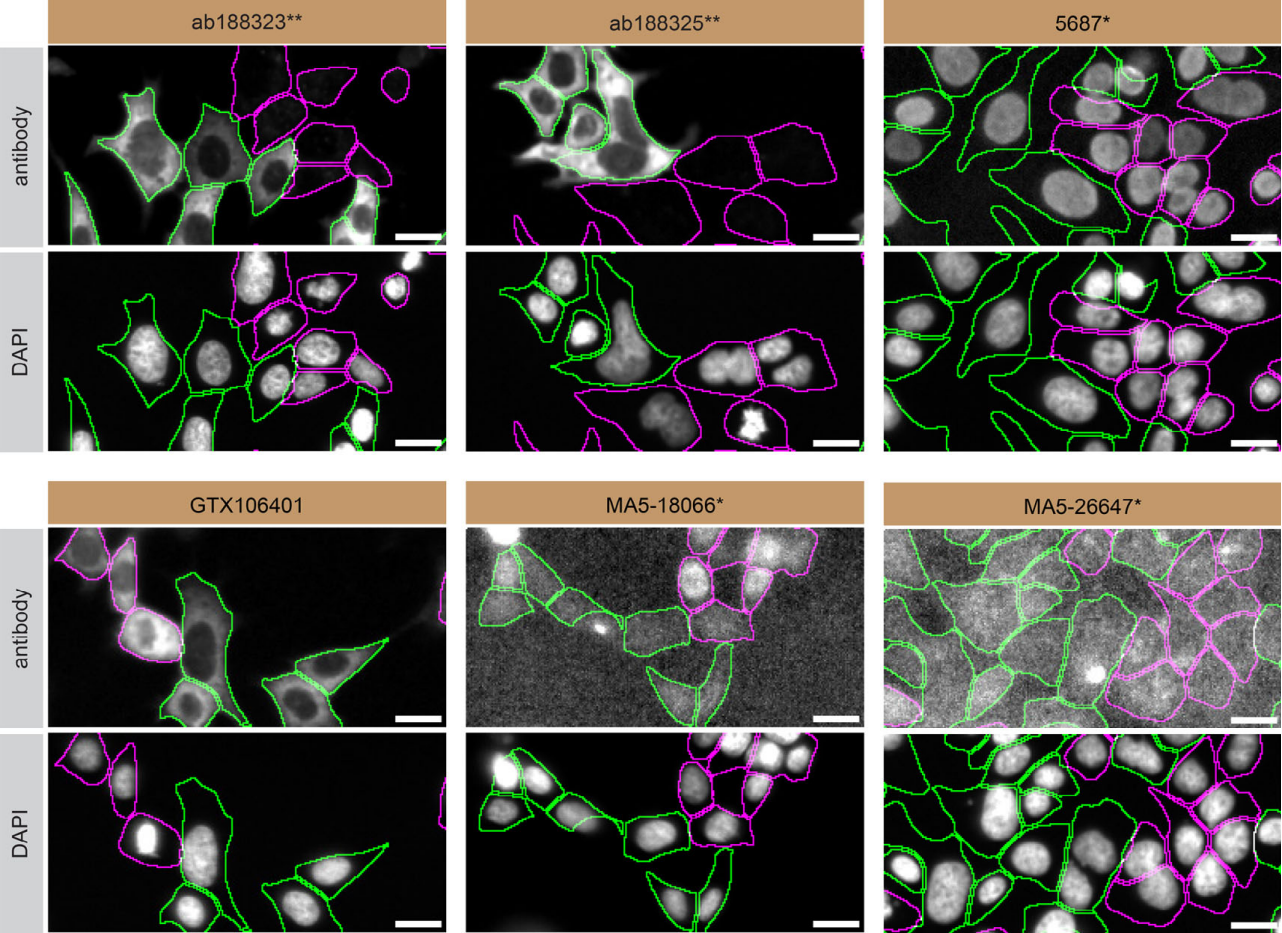
PPP2R5D antibody screening by immunofluorescence. HAP1 WT and *PPP2R5D* KO cells were labelled with a green or a far-red fluorescent dye, respectively. WT and KO cells were mixed and plated to a 1:1 ratio in a 96-well plate with optically clear flat-bottom. Cells were stained with the indicated PPP2R5D antibodies (first panel) and with the corresponding Alexa-fluor 555 coupled secondary antibody including DAPI (second panel). Acquisition of the blue (nucleus-DAPI), green (identification of WT cells), red (antibody staining) and far-red (identification of KO cells) channels was performed. Representative images of the merged blue and red (grayscale) channels are shown. WT cells were outlined with green dashed lines while KO cells were outlined with magenta dashed lines. When the concentration was not indicated by the supplier, which was the case for antibodies ab188325**, 5687*, GTX106401, MA5-18066* and MA5-26647*, we tested antibodies at the concentrations found below. At these concentrations, the signal from each antibody was in the range of detection of the microscope used. Antibody dilution used: ab188323** at 1/200, ab188325** at 1/300, 5687* at 1/70, GTX106401 at 1/1000, MA5-18066* at 1/1000, MA5-26647* at 1/1000. Bars = 10 μm. *Monoclonal antibody, **Recombinant antibody.

For immunofluorescence, antibodies were screened using a mosaic strategy, as per our standard procedure. First, HAP1 WT and *PPP2R5D* KO cell lines were labelled with different fluorescent dyes in order to distinguish the two cell lines, and the six PPP2R5D antibodies were evaluated. Cells were imaged in the same field of view to reduce staining, imaging and image analysis bias ( Figure 3). Quantification of immunofluorescence intensity in hundreds of WT and KO cells was performed for each antibody tested. The images presented in Figure 3 are representative of the results of this analysis.

In summary, we have screened six PPP2R5D commercial antibodies by Western Blot, immunoprecipitation and immunofluorescence. Several high-quality antibodies that successfully detect PPP2R5D under our standardized experimental conditions were identified. In our effort to address the antibody reliability and reproducibility challenges in scientific research, the authors recommend the antibodies that demonstrated to be underperforming under our standard procedure be removed from the commercial antibody market. However, the authors do not engage in result analysis or offer explicit antibody recommendations. A limitation of this study is the use of universal protocols - any conclusions remain relevant within the confines of the experimental setup and cell line used in this study. Our primary aim is to deliver top-tier data to the scientific community, grounded in Open Science principles. This empowers experts to interpret the characterization data independently, enabling them to make informed choices regarding the most suitable antibodies for their specific experimental needs. Guidelines on how to interpret the antibody characterization data in this study are available on the YCharOS Gateway. ^26^

The underlying data for this study can be found on Zenodo, an open-access repository for which YCharOS has its own collection of antibody characterization reports. ^27^^,^^28^

## Methods

### Antibodies

All PPP2R5D antibodies are listed in Table 2, together with their corresponding Research Resource Identifiers, or RRID, to ensure the antibodies are cited properly. ^29^ Peroxidase-conjugated goat anti-rabbit and anti-mouse antibodies are from Thermo Fisher Scientific (cat. number 65-6120 and 62-6520). Alexa-555-conjugated goat anti-rabbit and anti-mouse secondary antibodies are from Thermo Fisher Scientific (cat. number A21429 and A21424).

**Table 2.** Summary of the PPP2R5D antibodies tested.

Company	Catalog number	Lot number	RRID (Antibody Registry)	Clonality	Clone ID	Host	Concentration (μg/μL)	Vendors recommended applications
Abcam	ab188323 **	1023484-1	AB_3065198	recombinant- mono	EPR15617	rabbit	0.239	WB, IP, IF
Abcam	ab188325 **	GR169019-2	AB_3065199	recombinant- mono	EPR15617-50	rabbit	0.315	WB, IP
Cell Signaling Technology	5687 *	1	AB_10829041	monoclonal	H5D12	mouse	0.077	WB, IP
GeneTex	GTX106401	39694	AB_1241205	polyclonal	-	rabbit	1.0	WB
Thermo Fisher Scientific	MA5-18066 *	YE3914232	AB_2539449	monoclonal	H5D12	mouse	1.0	WB, IP
Thermo Fisher Scientific	MA5-26647 *	YE3914196	AB_2725096	monoclonal	OTI3B3	mouse	1.0	WB

Wb=Western Blot; IF= immunofluorescence; IP=immunoprecipitation.

* Monoclonal antibody.

** Recombinant antibody.

### Cell culture

Both HAP1 WT and *PPP2R5D* KO cell lines used are listed in Table 1, together with their corresponding RRID, to ensure the cell lines are cited properly. ^30^ Cells were cultured in DMEM high-glucose (GE Healthcare cat. number SH30081.01) containing 10% fetal bovine serum (Wisent, cat. number 080450), 2 mM L-glutamate (Wisent cat. number 609065, 100 IU penicillin and 100 μg/mL streptomycin (Wisent cat. number 450201).

### Antibody screening by Western Blot

HAP1 WT and *PPP2R5D* KO were collected RIPA buffer (25 mM Tris-HCl pH 7.6, 150 mM NaCl, 1% NP-40, 1% sodium deoxycholate, 0.1% SDS) from Thermo Fisher Scientific (cat. number 89901) supplemented with 1× protease inhibitor cocktail mix (MilliporeSigma, cat. number P8340). Lysates were sonicated briefly and incubated for 30 min on ice. Lysates were spun at ~110,000 × g for 15 min at 4°C and equal protein aliquots of the supernatants were analyzed by SDS-PAGE and Western Blot. BLUelf prestained protein ladder from GeneDireX (cat. number PM008-0500) was used.

Western Blots were performed with precast midi 4-20% Tris-Glycine polyacrylamide gels from Thermo Fisher Scientific (cat. number WXP42012BOX) ran with Tris/Glycine/SDS buffer from bio-Rad (cat. number 1610772), loaded in Laemmli loading sample buffer from Thermo Fisher Scientific (cat. number AAJ61337AD) and transferred on nitrocellulose membranes. Proteins on the blots were visualized with Ponceau S staining (Thermo Fisher Scientific, cat. number BP103-10) which is scanned to show together with individual Western Blot. Blots were blocked with 5% milk for 1 hr, and antibodies were incubated overnight at 4°C with 5% milk in TBS with 0.1% Tween 20 (TBST) (Cell Signalling Technology, cat. number 9997). Following three washes with TBST, the peroxidase conjugated secondary antibody was incubated at a dilution of ~0.2 μg/mL in TBST with 5% milk for 1 hr at room temperature followed by three washes with TBST. Membranes were developed with Pierce ECL from Thermo Fisher Scientific (cat. number 32106) Or Clarity Western ECL Substrate from Bio-Rad (cat. number 1705061) prior to detection with the iBright™ CL1500 Imaging System from Thermo Fisher Scientific (cat. number A44240).

### Antibody screening by immunoprecipitation

Antibody-bead conjugates were prepared by adding 2 μg of antibody at an unknown concentration to 500 μL of Pierce IP Lysis Buffer from Thermo Fisher Scientific (cat. number 87788) in a 1.5 mL microcentrifuge tube, together with 30 μL of Dynabeads protein A - (for rabbit antibodies) or protein G - (for mouse) from Thermo Fisher Scientific (cat. number 10002D and 10004D, respectively). Tubes were rocked for ~1 hr at 4°C followed by two washes to remove unbound antibodies.

HAP1 WT were collected in Pierce IP buffer (25 mM Tris-HCl pH 7.4, 150 mM NaCl, 1 mM EDTA, 1% NP-40 and 5% glycerol) supplemented with protease inhibitor. Lysates were rocked 30 min at 4°C and spun at 110,000 × g for 15 min at 4°C. 0.5 mL aliquots at 2.0 mg/mL of lysate were incubated with an antibody-bead conjugate for ~1 hr at 4°C. The unbound fractions were collected, and beads were subsequently washed three times with 1.0 mL of IP lysis buffer and processed for SDS-PAGE and Western Blot on a precast midi 4-20% Tris-Glycine polyacrylamide gels. VeriBlot for IP Detection Reagent:HRP (Abcam, cat. number ab131366) was used as a secondary detection system at a concentration of 0.1 μg/mL.

### Antibody screening by immunofluorescence

HAP1 WT and *PPP2R5D* KO were labelled with a green and a far-red fluorescence dye, respectively. The fluorescent dyes used are from Thermo Fisher Scientific (cat. number C2925 and C34565). The nuclei were labelled with DAPI (Thermo Fisher Scientific, cat. Number D3571) fluorescent stain. WT and KO cells were then plated in a 96-well plate with optically clear flat-bottom as a mosaic and incubated for 24 hrs in a cell culture incubator at 37 ^o^C, 5% CO _2_. Cells were fixed in 4% paraformaldehyde (PFA) (Beantown chemical, cat. number 140770-10 ml) in phosphate buffered saline (PBS) (Wisent, cat. number 311-010-CL). Cells were permeabilized in PBS with 0.1% Triton X-100 (Thermo Fisher Scientific, cat. number BP151-500) for 10 min at room temperature and blocked with PBS with 5% bovine serum albumin (BSA) (Wisent, cat. number 800-095), 5% goat serum (Gibco, cat. number 16210-064) and 0.01% Triton X-100 for 30 min at room temperature. Cells were incubated with IF buffer (PBS, 5% BSA, 0,01% Triton X-100) containing the primary PPP2R5D antibodies overnight at 4°C. Cells were then washed 3 × 10 min with IF buffer and incubated with corresponding Alexa Fluor 555-conjugated secondary antibodies in IF buffer at a dilution of 1.0 μg/mL for 1 hr at room temperature with DAPI. Cells were washed 3 × 10 min with IF buffer and once with PBS.

Images were acquired on an ImageXpress micro widefield high-content microscopy system (Molecular Devices), using a 20× NA 0.95 water objective lens and scientific CMOS camera (16-bit, 1.97 mm field of view), equipped with 395, 475, 555 and 635 nm solid state LED lights (Lumencor Aura III light engine) and bandpass emission filters (432/36 nm, 520/35 nm, 600/37 nm and 692/40 nm) to excite and capture fluorescence emission for DAPI, CellTracker ^TM^ Green, Alexa fluor 555 and CellTracker ^TM^ Red, respectively. Images had pixel sizes of 0.68 × 0.68 microns. Exposure time was set with maximal (relevant) pixel intensity ~80% of dynamic range and verified on multiple wells before acquisition. Since the IF staining varied depending on the primary antibody used, the exposure time was set using the most intensely stained well as reference. Frequently, the focal plane varied slightly within a single field of view. To remedy this issue, a stack of three images per channel was acquired at a z-interval of 4 microns per field and best focus projections were generated during the acquisition (MetaExpress v6.7.1, Molecular Devices). Segmentation was carried out on the projections of CellTracker ^TM^ channels using CellPose v1.0 on green (WT) and far-red (KO) channels, using as parameters the ‘cyto’ model to detect whole cells, and using an estimated diameter tested for each cell type, between 15 and 20 microns. ^31^ Masks were used to generate cell outlines for intensity quantification. Figures were assembled with Adobe Photoshop (version 24.1.2) to adjust contrast then assembled with Adobe Illustrator (version 27.3.1).

## Data Availability

Zenodo: Antibody Characterization Report for PPP2R5D, https://doi.org/10.5281/zenodo.10108248. ^27^ Zenodo: Dataset for the PPP2R5D antibody screening study, https://doi.org/10.5281/zenodo.10119578. ^28^ Data are available under the terms of the Creative Commons Attribution 4.0 International license (CC-BY 4.0).
