# Supplementary material for: A guide to selecting high-performing antibodies for Serine/threonine-protein phosphatase 2A 56 kDa regulatory subunit delta isoform (PPP2R5D) for use in Western Blot, immunoprecipitation and immunofluorescence
Source: F1000Res. 2024 Jul 9;13:1. Originally published 2024 Jan 3. [Version 2] doi: 10.12688/f1000research.145146.2 (PMC11811605; doi:10.12688/f1000research.145146.2)
Supplement: Peer review history

# Reviewer report: Reviewer response for version 2

**DOI:** 10.5256/f1000research.168890.r300981

**Published:** 2024-08-07

**Authors:** Nadia Bouhamdani

**Affiliations:** 1 Medical Genetics Department & Department of Chemistry and Biochemistry, Vitalité Health Network, Dr Georges-L.-Dumont University Hospital Center, Université de Sherbrooke, Université de Moncton, Moncton, New Brunswick, Canada

The revisions by the authors are sufficient.

Are sufficient details of methods and materials provided to allow replication by others?

Yes

Is the rationale for creating the dataset(s) clearly described?

Yes

Are the datasets clearly presented in a useable and accessible format?

Yes

Are the protocols appropriate and is the work technically sound?

Partly

Reviewer Expertise:

Cancer research, proteomics, functional genomics, rare disease research.

I confirm that I have read this submission and believe that I have an appropriate level of expertise to confirm that it is of an acceptable scientific standard.

# Reviewer report: Reviewer response for version 2

**DOI:** 10.5256/f1000research.168890.r303543

**Published:** 2024-07-30

**Authors:** Dario Siniscalco, Mauro Finicelli

**Affiliations:** 1 University of Campania, Naples, Italy

- It would be interesting to see also other cell lines (i.e. nervous system derived cells or tissues).

 - It would be more informative if the authors had used 4 cell lines for their experiments. This is to say, the use of a highly-expressing PPP2R5D cell line, a normal-expressing PPP2R5D cell line, a low-expressing cell line and a KO cell line. The combination of these results could give a valid overview about the effectiveness of the antibodies tested in all the assays. 

 - Although the data from DepMap transcriptomics database about the expression levels of PPP2R5D in HAP1 cells and KO ones, it could be useful a check of the authors in their cell lines. The authors should test the effective levels on PPP2R5D mRNA levels in both the cell lines used for their experiments.

 - One of the ab used has been discontinued by the company. Authors should consider this in discussion section.

 - It could be useful to add a direct link to the ab's vendor webpage in Table 2, as the antibody registry.org only has a link to the main vendor's webpage and not to the specific ab's webpage.

 - Methods, WB: two different ECL systems have been employed. This could affect signal detection. Briefly explain.

 - ICC: the authors must indicate the rationale used for identification of the antibody dilution applied in ICC experiments. E.g., although for MA5-18066 the antibody dilution recommended in the brochure is assay-dependent in HeLa cells the antibody was diluted 1:100 with a good nuclear signal; why did the authors used 1:1000? Moreover, it is not clearly reported if the authors used a negative control (i.e., reaction without primary antibody) for each experiment.

 - About ICC experiments, the authors should provide a single image for PPP2R5D expression levels in HAP1 and KO cells, respectively, to corroborate the results obtained in co-cultured cells. I appreciate if the authors could provide the single channel and merged images representative of the experiments.

Are sufficient details of methods and materials provided to allow replication by others?

Partly

Is the rationale for creating the dataset(s) clearly described?

Yes

Are the datasets clearly presented in a useable and accessible format?

Yes

Are the protocols appropriate and is the work technically sound?

Yes

Reviewer Expertise:

autism; nervous system; gene expression; protein levels; enzyme activity; cell biology; miRNA.

We confirm that we have read this submission and believe that we have an appropriate level of expertise to confirm that it is of an acceptable scientific standard.

# Response

**Published:** 2024-12-12

**Authors:** Carl Laflamme

**Affiliations:** McGill University, Canada

Dear Dr. Siniscalco,

 Thank you for your feedback on the PPP2R5D antibody characterization data note.

 The authors of this study are antibody experts, though not specialists on the PPP2R5D protein itself. The primary objective of this work is to provide an unbiased evaluation of the performance of currently available PPP2R5D antibodies. The processes and methodologies employed in this study were endorsed by 12 antibody manufacturers and disease foundations. Additionally, the protocols have been shared as a preprint and are accepted for publication in *Nature Protocols*, with an expected release by the end of 2024.

 As demonstrated by DepMap, which provides RNA sequencing data for thousands of cancer cell lines, PPP2R5D is ubiquitously expressed. The antibody ab188323 has been previously validated in western blot analysis by Abcam using various cell lines, including A431, HeLa, and MCF7, showing a similar pattern to our findings in HAP1 cells. We acknowledge that RNA and protein levels may not always correlate perfectly. However, we confirm that HAP1 expresses adequate PPP2R5D protein levels as a subset of antibodies successfully detected PPP2R5D, as evidenced by the lack of signal in knockout (KO) lysates.

 The polyclonal antibody GTX106401 has been discontinued, which is a common occurrence with polyclonal products due to their finite nature. This underscores the importance of the YCharOS initiative and its industry partners focusing on recombinant and monoclonal antibodies. Of the six antibodies tested in this study, five fall into these categories.

 Regarding your specific concerns:

**1) Antibody detection sensitivity:**

 Our standard protocol begins with the Pierce ECL system using the manufacturer-recommended antibody dilutions. For antibodies yielding weak signals, we increase the concentration fivefold and switch to the Bio-Rad ECL enhanced system, which has provided stronger signals in our experience.

**2) ICC specificity testing:**

 In ICC experiments, antibodies were evaluated using a mosaic of wild-type (WT) and KO cell lines to assess specificity. For example, MA5-18066 showed similar staining in both WT (green outline) and KO (pink outline) cell lines, indicating it is unsuitable under the tested conditions. However, two antibodies demonstrated specific cytoplasmic staining, clearly observed by the lack of signal in KO cells, with no nuclear signal.

 Testing antibody performance in WT and KO cell lines within the same field of view represents the gold standard in antibody characterization. This approach minimizes user and imaging biases and ensures that identical microscopy parameters are applied to both conditions, enhancing the reliability of the results.

**3) Links from the Antibody Registry to vendor websites:**

 I contacted the Antibody Registry to address your concern, and they confirmed that they maintain updated links to the direct product pages for all companies that provide them. Unfortunately, not all companies supply these updates. The current Table 2 provides all the necessary information to trace the antibodies used in this study.

 We hope this addresses your feedback comprehensively.

 Thank you once again for your valuable input.

 Best regards,

 Carl Laflamme

# Response

**Published:** 2024-12-16

**Authors:** Dario Siniscalco

**Affiliations:** Experimental Medicine, University of Campania Vanvitelli, Naples, IT, Italy

Authors succesfully answered to my comments.

# Reviewer report: Reviewer response for version 1

**DOI:** 10.5256/f1000research.159050.r269488

**Published:** 2024-05-20

**Authors:** Nadia Bouhamdani

**Affiliations:** 1 Medical Genetics Department & Department of Chemistry and Biochemistry, Vitalité Health Network, Dr Georges-L.-Dumont University Hospital Center, Université de Sherbrooke, Université de Moncton, Moncton, New Brunswick, Canada

The authors characterize six commercially available antibodies for PPP2R5D using a standardized experimental protocol based on comparing read-outs in knockout cell lines and isogenic parental controls. The main objective of this study is to provide a published report to guide researchers in the field in selecting the most appropriate available antibodies for their specific research needs.

 In general, this data note article is clear and straight forward; however, important details are lacking. Here are some comments/questions that need to be addressed: 

1.  In the section ‘ *results and discussion*’, the authors mention that cell models used to characterize PPP2R5D antibodies were chosen based on analyzing ‘DepMap transcriptomics database’ and cell lines expressing mRNA levels of PPP2R5D greater than 2.5log _2_(TPM +1) were deemed suitable for experiments. Does this mean that the only cell line identified with this cut off was HAP1? In my opinion, an important limitation of this study is to have only tested one pair of isogenic models (WT vs KO). Please elaborate. At a minimum, this should be mentioned as a limitation.
2. Why only consider cancer cell models? Have the authors considered using other types of cells (patient cells or other?) where expression levels of PPP2R5D vary. This would be important to analyze/highlight antibody sensitivity. 
3. Researchers in the field working on PPP2R5D-related neurodevelopmental disorder (MRD35/Jordan’s syndrome) often work with patient cells or other models that better recapitulate MRD35 disease characteristics (PPP2R5D pathogenic variants). Have these 6 antibodies been tested on PPP2R5D mutated cell lines (genetic variants, SNP vs. KO)? If so, do these antibodies recognize mutated PPP2R5D and if not, it may also be important to mention the importance of this in the limitation section. 
4. The 6 antibodies - how were they chosen? Are these 6 antibodies the only commercially available? 
5. An important aspect of using high-quality and specific antibodies in research is assuring that the antibody selected does not cross-react with other known isoforms. Have these antibodies been tested for cross-reactivity with other PP2A-B56 family isoforms (e.g., PPP2R5C, PPP2R5B, etc.)? 
6. I would suggest presenting Table 2 (antibody selection) after Table 1 in the Results and discussion section (before western blot and Immunofluorescence results).
7. In the IP experiments (Figure 2) –Could the authors please expand on why we see a multiple band pattern in Figure 2. Earlier in the manuscript, the authors suggest that different MWs can be observed for PPP2R5D depending on lysis buffer used. However, in my understanding, only the IP lysis buffer was used for IP experiments suggesting that PPP2R5D band should be around 75KDa. Is the multiple band pattern only seen when using gradient gels (4-20%)? And if so, it would be important to elaborate which band corresponds to PPP2R5D as some antibodies enrich bottom bands, and others, top bands. Please elaborate on non-specificity or possible cleavage products/degradation as this does not seem to have been tested. 
8. In the Discussion section, it would be important to clearly summarize which of the 6 antibodies the authors identified as high-quality antibodies that successfully detect PPP2R5D (and which ones are more suitable for protocols tested (WB vs. IP vs. IF)) as this crucial information is missing. 

Are sufficient details of methods and materials provided to allow replication by others?

Yes

Is the rationale for creating the dataset(s) clearly described?

Yes

Are the datasets clearly presented in a useable and accessible format?

Yes

Are the protocols appropriate and is the work technically sound?

Partly

Reviewer Expertise:

Cancer research, proteomics, functional genomics, rare disease research.

I confirm that I have read this submission and believe that I have an appropriate level of expertise to confirm that it is of an acceptable scientific standard, however I have significant reservations, as outlined above.

# Response

**Published:** 2024-07-05

**Authors:** Kathleen Southern

**Affiliations:** McGill University, Canada

Dear Nadia Bouhamdani, thank you for your comprehensive review of the article “A guide to selecting high-performing antibodies for Serine/threonine-protein phosphatase 2A 56 kDa regulatory subunit delta isoform (PPP2R5D) for use in Western Blot, immunoprecipitation and immunofluorescence”. We appreciate your feedback and commitment to enhancing the reproducibility and comprehension of the antibody characterization data provided in the article. Please see below our responses to your comments/questions. A new version of the article will be submitted shortly after to address a few of your concerns.

1. ** In the section ‘ *results and discussion*’, the authors mention that cell models used to characterize PPP2R5D antibodies were chosen based on analyzing ‘DepMap transcriptomics database’ and cell lines expressing mRNA levels of PPP2R5D greater than 2.5log _2_(TPM +1) were deemed suitable for experiments. Does this mean that the only cell line identified with this cut off was HAP1? In my opinion, an important limitation of this study is to have only tested one pair of isogenic models (WT vs KO). Please elaborate. At a minimum, this should be mentioned as a limitation. **
  Response: From analyzing the public expression database on Depmap, we found that HAP1 expressed PPP2R5D transcript at 5.7 log _2_ (TPM+1), which is significantly above the minimum threshold level we’ve established. From the results, it is evident that the selection of HAP1 as cell line for antibody testing was appropriate, as PPP2R5D was easily detected in the lysates when testing the antibodies by western blot. We acknowledge that antibody characterization data generated by following the described protocols does not provide a comprehensive evaluation of the tested antibodies. However, the strength of our approach lies in the simultaneous testing of multiple antibodies from a variety of commercial sources using KO cell lines, aimed at identifying the most suitable antibodies for specific applications. A detailed description of the orthogonal strategy applied to select cell lines for the study is outlined in our antibody characterization platform, available on Nature Protocols (DOI: 10.21203/rs.3.pex-2607/v1).

**2. Why only consider cancer cell models? Have the authors considered using other types of      cells (patient cells or other?) where expression levels of PPP2R5D vary. This would be important to analyze/highlight antibody sensitivity.**

**3. Researchers in the field working on PPP2R5D-related neurodevelopmental disorder (MRD35/Jordan’s syndrome) often work with patient cells or other models that better recapitulate MRD35 disease characteristics (PPP2R5D pathogenic variants). Have these 6 antibodies been tested on PPP2R5D mutated cell lines (genetic variants, SNP vs. KO)? If so, do these antibodies recognize mutated PPP2R5D and if not, it may also be important to mention the importance of this in the limitation section**. 

 Response to point 2 & 3: We understand that mutations in the epitope coding sequence or elsewhere in the gene coding for the intended target could affect antibody binding, although we think this would be a rare occurrence. However, this is an inherent limitation of any approach that uses cancer cell lines. We have addressed this limitation in the summary paragraph of the results and discussion section.

**4. The 6 antibodies - how were they chosen? Are these 6 antibodies the only commercially available?**

 Response: The antibodies to be tested are donated by YCharOS partners ( https://ycharos.com/partners/), who have access to 80% of renewable antibodies covered in commercial catalogs, the other 20% of antibodies from sources that are not partners, why is why we don’t test them. When the partners are informed of upcoming targets, they donate the antibodies they have the capacity to supply at the time the study commences.

**5. An important aspect of using high-quality and specific antibodies in research is assuring that the antibody selected does not cross-react with other known isoforms. Have these antibodies been tested for cross-reactivity with other PP2A-B56 family isoforms (e.g., PPP2R5C, PPP2R5B, etc.)?**

 Response: As indicated on their catalog pages, these antibodies are intended to target PPP2R5D, which is why we have tested them, side-by-side, using a *PPP2R5D *KO cell line. This allows viewers to determine whether the antibodies are detecting PPP2R5D or identify certain variants which they can then use to further validate for cross-reactivity with other PP2A-B56 isoforms. Unfortunately, this degree of validation is outside the scope of our study but we encourage experts in the field to utilize the validation data provided as a starting point to enhance their PPP2R5D related research.

**6. I would suggest presenting Table 2 (antibody selection) after Table 1 in the Results and discussion section (before western blot and Immunofluorescence results).**

 Response: Thank you for bringing up this concern. The newly submitted version of this article will present Table 2 before presenting the results.

           7 **. In the IP experiments (Figure 2) –Could the authors please expand on why we see a multiple band pattern in Figure 2. Earlier in the manuscript, the authors suggest that different MWs can be observed for PPP2R5D depending on lysis buffer used. However, in my understanding, only the IP lysis buffer was used for IP experiments suggesting that PPP2R5D band should be around 75KDa. Is the multiple band pattern only seen when using gradient gels (4-20%)? And if so, it would be important to elaborate which band corresponds to PPP2R5D as some antibodies enrich bottom bands, and others, top bands. Please elaborate on non-specificity or possible cleavage products/degradation as this does not seem to have been tested.**

 Response: When carrying out the IP experiment (Figure 2), the lysates were freshly prepared and antibodies were immediately processed for IP, which was not the case for the lysis buffer comparison experiment (Figure 1B). In Figure 1, frozen lysates were used to carry out the experiments (A and B). This can explain the difference in signal pattern seen in Figure 2 vs Figure 1B.

**8. In the Discussion section, it would be important to clearly summarize which of the 6 antibodies the authors identified as high-quality antibodies that successfully detect PPP2R5D (and which ones are more suitable for protocols tested (WB vs. IP vs. IF)) as this crucial information is missing.**

 Response: As described in the final paragraphs of the results and discussion section, the authors do not participate in results analysis nor offer explicit antibody recommendations, therefore preventing them from identifying which antibodies were high-performing in each respective application. Due to the confines of the experimental setup, the factors that can influence the performance of antibodies and cell line used, we do not draw conclusions. We leave this analysis up to the viewers and within our gateway we feature an editorial that can be used as a guide on how the scientific community can utilize and analyze the YCharOS data to identify high-performing antibodies within the confines of our protocol (DOI: 10.12688/f1000research.141719.1).

 Furthermore, given that that the article is formatted as a Data Note, it does not require the results to be discussed or concluded.
